# β1600 Q.Clear Digital Reconstruction of [^68^Ga]Ga-DOTANOC PET/CT Improves Image Quality in NET Patients

**DOI:** 10.3390/jcm13133841

**Published:** 2024-06-29

**Authors:** Martina Di Franco, Emilia Fortunati, Lucia Zanoni, Norma Bonazzi, Cristina Mosconi, Claudio Malizia, Simona Civollani, Davide Campana, Elisa Andrini, Giuseppe Lamberti, Vincenzo Allegri, Stefano Fanti, Valentina Ambrosini

**Affiliations:** 1 Nuclear Medicine, Alma Mater Studiorum, University of Bologna, 40126 Bologna, Italy; norma.bonazzi@studio.unibo.it (N.B.); stefano.fanti@aosp.bo.it (S.F.); valentina.ambrosini@unibo.it (V.A.); 2 Nuclear Medicine, IRCCS, Azienda Ospedaliero-Universitaria di Bologna, 40138 Bologna, Italy; lucia.zanoni@aosp.bo.it (L.Z.); claudio.malizia@aosp.bo.it (C.M.); simona.civollani@aosp.bo.it (S.C.); vincenzo.allegri@aosp.bo.it (V.A.); 3 Department of Medical and Surgical Sciences (DIMEC), Alma Mater Studiorum, University of Bologna, 40138 Bologna, Italy; cristina.mosconi@aosp.bo.it (C.M.); davide.campana@unibo.it (D.C.); elisa.andrini3@unibo.it (E.A.); giuseppe.lamberti8@unibo.it (G.L.); 4 Department of Radiology, IRCCS, Azienda Ospedaliero-Universitaria di Bologna, 40138 Bologna, Italy; 5 Medical Oncology, IRCCS, Azienda Ospedaliero-Universitaria di Bologna, 40138 Bologna, Italy

**Keywords:** Q.Clear, PET/CT, [^68^Ga]Ga-DOTANOC, neuroendocrine neoplasms, neuroendocrine tumors, NET

## Abstract

**Background:** Image reconstruction is crucial for improving overall image quality and diagnostic accuracy. Q.Clear is a novel reconstruction algorithm that reduces image noise. The aim of the present study is to assess the preferred Q.Clear β-level for digital [^68^Ga]Ga-DOTANOC PET/CT reconstruction vs. standard reconstruction (STD) for both overall scan and single-lesion visualization. **Methods:** Inclusion criteria: (1) patients with/suspected neuroendocrine tumors included in a prospective observational monocentric study between September 2019 and January 2022; (2) [^68^Ga]Ga-DOTANOC digital PET/CT and contrast-enhanced-CT (ceCT) performed at our center at the same time. Images were reconstructed with STD and with Q.Clear β-levels 800, 1000, and 1600. Scans were blindly reviewed by three nuclear-medicine experts: the preferred β-level reconstruction was independently chosen for the visual quality of both the overall scan and the most avid target lesion < 1 cm (t) and >1 cm (T). PET/CT results were compared to ceCT. Semiquantitative analysis was performed (STD vs. β1600) in T and t concordant at both PET/CT and ceCT. Subgroup analysis was also performed in patients presenting discordant t. **Results**: Overall, 52 patients were included. β1600 reconstruction was considered superior over the others for both overall scan quality and single-lesion detection in all cases. The only significantly different (*p* < 0.001) parameters between β1600 and STD were signal-to-noise liver ratio and standard deviation of the liver background. Lesion-dependent parameters were not significantly different in concordant T (*n* = 37) and t (*n* = 10). Among 26 discordant t, when PET was positive, all findings were confirmed as malignant. **Conclusions**: β1600 Q.Clear reconstruction for [^68^Ga]Ga-DOTANOC imaging is feasible and improves image quality for both overall and small-lesion assessment.

## 1. Introduction

Technical improvements to Positron Emission Tomography with Computed Tomography (PET/CT) have focused on obtaining better image quality through the improvement of image contrast and the minimization of noise level. Image reconstruction is crucial for improving overall image quality and diagnostic accuracy [1].

A widely used image reconstruction algorithm, OSEM, is based on the repetition of a convergence algorithm that translates PET coincidence data into an image. Iterating the function, a new image is produced. This reconstruction model is affected by increased noise when the convergence is full; therefore, it is stopped after 2–4 iterations. Scarce iterations can lower OSEM quantitative accuracy and misrepresent small lesions.

A Bayesian penalized-likelihood image reconstruction algorithm, named Q.Clear, has recently been implemented for digital PET/CT tomographs (GE Healthcare) to improve quantification accuracy and image quality [2]. Q.Clear reconstruction incorporates a noise-suppression term called “relative difference penalty”, for which the formula is available at: https://www.gehealthcare.com (accessed on 18 June 2024). Through regularization, the algorithm reaches full convergence without the need to stop iterations, resulting in preserving the edges and lowering the background at every voxel. The strength of the regularizing term is controlled by a “β” variable. The selection of the best optimal penalization factor (β-level) regulates the penalty term in the reconstruction algorithm with a consecutive variability in signal recovery and background. Lower β-levels are generally associated with higher noise and sharp contrast, while higher β-levels suppress image noise with the possibility of excessive smoothing [3].

It is well known that Q.Clear reconstruction improves overall image quality in comparison to other reconstruction algorithms by improving contrast recovery and noise suppression, therefore resulting in better lesion detectability [4,5,6,7,8,9].

Small-lesion detection is both a challenging and clinically relevant issue: Q.Clear is expected to improve small-lesion detectability by reducing image noise [10,11]. However, this setting has not been extensively studied, and there is no consensus on the best β-level to employ.

To date, most literature data has investigated the utility of Q.Clear on [^18^F]F-FDG (Fluorodeoxyglucose) images. Some analyses conducted on phantoms have shown that Q.Clear reconstruction seems to detect sub-centimetric findings better than OSEM reconstruction [12,13].

Regarding the particular setting of small lesions and their characterization, neuroendocrine neoplasms (NEN) could benefit from Q.Clear reconstruction. [^68^Ga]Ga-DOTA-peptide PET/CT is the gold standard for imaging well-differentiated neuroendocrine tumors (NET, with high expression of somatostatin receptors—SST), mainly for staging, assessing SST status, selecting patients for Peptide Receptor Radionuclide Therapy (PRRT), and monitoring the response to therapy [14,15,16].

[^68^Ga]Ga-DOTA-peptide PET/CT shows high sensitivity and specificity for the diagnosis of NET [17,18,19,20,21]. The most common site of NET metastases is the liver (up to 85% of patients), and its involvement is associated with reduced survival. However, the physiological moderate-to-intense and often heterogeneous [^68^Ga]Ga-DOTA-peptide uptake at the liver level limits the detection of metastases [17,18]. For extra-hepatic metastasis detection (e.g., bone [19] and node [20]), [^68^Ga]Ga-DOTA-peptide PET/CT resulted in more accuracy than contrast-enhanced diagnostic CT (ceCT).

Q.Clear reconstruction may be crucial for detecting small lesions at both liver and extra-liver levels, with potential impact on patient management.

The literature about Q.Clear utility in NET patients is still very limited, and no definitive β-levels have been reported.

The aim of the present study was to assess the preferred Q.Clear β-level for digital [^68^Ga]Ga-DOTANOC PET/CT reconstruction vs. standard reconstruction (STD) for both overall scan quality and single-lesion visualization (for the most avid finding < 1 cm and >1 cm, respectively).

## 2. Materials and Methods

Among the patients with NET or suspected NET who underwent [^68^Ga]Ga-DOTANOC PET/CT at our center and were included in a prospective monocentric CE-approved electronic archive (131/2017/O/Oss), those meeting the following criteria were included in the analysis: (1) [^68^Ga]Ga-DOTANOC PET/CT performed on a digital tomograph (GE MI) between September 2019 and January 2022 and ceCT performed at our center at the same time; (2) no significant radiotracer extravasation.

[^68^Ga]Ga-DOTANOC PET/CT was acquired on a digital tomograph according to standard practice and following EANM guidelines (100–200 MBq, uptake time 60 min, 3 min per bed position) [14,15]. Overall, 52 patients were included. Areas of increased uptake outside the tracer’s biodistribution were interpreted as positive (excluding areas of clearly benign/inflammatory findings). Images were reconstructed with standard OSEM (8 subsets, 4 iterations, 6 mm filter) + time of flight (STD) and Q.Clear algorithms with three different βlevels (800, 1000, and 1600) [22].

Scans were reviewed by three expert nuclear-medicine readers, unaware of the clinical data, who independently chose the preferred reconstruction (STD vs. β800 vs. β1000 vs. β1600) for the visual quality of both overall scan and single lesions (for the most avid target finding < 1 cm and >1 cm, respectively). Agreement among readers was assessed to define the best β-level reconstruction, as previously published. PET/CT results were compared to ceCT and revised by one expert radiologist.

Semiquantitative analysis (GE software AW server) of the most avid lesion < 1 cm (t) and > 1 cm (T) concordant at both PET/CT and ceCT was performed both on STD and the best β-level reconstruction: SUVmax, SUVmean, and standard deviation (SD) of, respectively, the most avid target lesion (T and t) and liver background (L); SUVmax-T/SUVmean-L; SUVmax-t/SUVmean-L; signal-to-noise liver ratio (SNR-L = SUVmean /SD); contrast-to-noise ratio (CNR = SUVmean (T or t)-SUVmeanSurroundingBackground/SDBackground). Note that a 5-centimeter-diameter ROI on disease-free liver parenchyma was used to measure liver background uptake.

A subgroup analysis was also performed in patients presenting small-sized discordant lesions on PET/CT and ceCT.

## 3. Statistical Analysis

All collected data were analyzed using R software version 4.1.0, with a significance level set at 0.05. The Friedman test was applied to semiquantitative parameters. In the case of significant results, post hoc analysis was performed using pair-by-pair Wilcoxon signed-rank tests.

## 4. Results

Overall, 52 patients with NET or suspected NET were included (M:F = 31:21; age: mean = 60.3 yo, median = 62.5 [52.7–73.0] yo). NET primary tumor sites were ileum (15/52, 29%), pancreas (11/52, 21%), other gastrointestinal (6/52, 11%), lung (4/52, 8%), and unknown (4/52,8%). A total of 12 of 52 (23%) were patients with suspected NET.

Indications to [^68^Ga]Ga-DOTANOC PET/CT were pre-treatment staging (7/52, 14%), post-surgical staging (6/52, 11%), evaluation of PRRT eligibility (2/52, 4%), suspected relapse (4/52, 8%), assessment during treatment (8/52, 15%), restaging after therapy (10/52, 19%), follow-up (2/52, 4%), localization of unknown primary (2/52, 4%) and for suspected NET (11/52, 21%) [Table 1].

Overall, [^68^Ga]Ga-DOTANOC PET/CT was positive in 37/52 patients (71%) and negative in 15/52 (29%), while ceCT was positive in 45/52 (87%) and negative in 7/52 (13%) patients.

**Figure 1:**
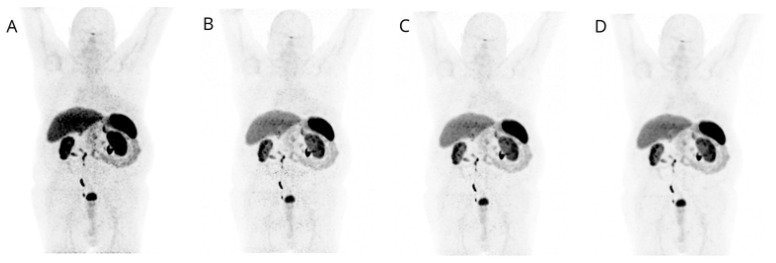
MIP images corresponding to STD (**A**), β-level 800 (**B**), β-level 1000 (**C**), and β-level 1600 (**D**) are displayed: lower image noise is evident in D, confirming the preferred image quality with β-level 1600.

**Figure 2:**
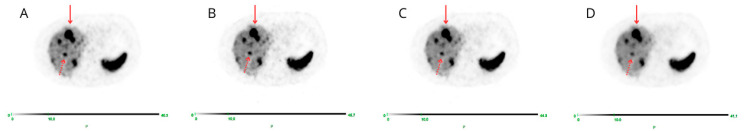
Transaxial PET images corresponding to STD (**A**), β-level 800 (**B**), β-level 1000 (**C**), and β-level 1600 (**D**) are displayed: both T (red arrow) and t (dashed arrow) are better appreciated on the D image corresponding to β-level 1600.

Visual image quality of PET β-1600 reconstruction was considered superior over the others (STD; β-800; β-1000) for both overall scan quality and single-lesion detection in all cases (52/52, 100%), with full agreement between the three readers (100%) (Figure 1 and Figure 2).

**Figure 3:**
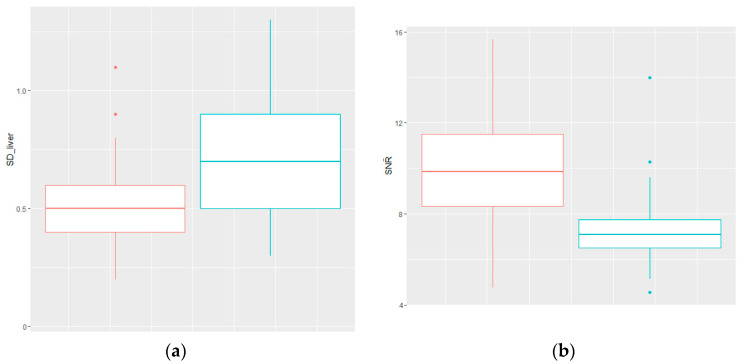
(**a**) Standard deviation of the liver background of β1600 (red) and STD (blue) are significantly different *p* < 0.001; (**b**) Signal-to-noise liver ratio of β1600 (red) and STD (blue) are significantly different at *p* < 0.001.

The only significantly different (*p* < 0.001) parameters between β1600 and STD (*p* < 0.001) were signal-to noise liver ratio (SNR-L) (β1600 vs. STD: mean = 9.9 vs. 7.3; median = 9.9 vs. 7.1; range: 4.7–15.7 vs. 4.6–14.0) and standard deviation of the liver background (β1600 vs. STD: mean = 0.5 vs. 0.7; median =0.5 vs. 0.7; range: 0.2–1.1 vs. 0.3–1.3) (Figure 3).

When lesion-dependent parameters (SUVmax, SUVmean, and CNR) were measured in the most avid lesion (>1 cm, T; n = 37), there were no statistically significant differences between β1600 and STD. This was also confirmed when lesion-dependent parameters were measured in the most avid concordant small lesion (<1 cm, t; n = 10) (Table 2).

In the subgroup of patients presenting small-sized (<1 cm) discordant lesions (n = 26) between PET/CT and ceCT, PET was negative in 13 cases and positive in the remaining half of the cases (13/26).

Among patients with PET-negative small discordant lesions (n = 13/26), in 4 cases, ceCT detected millimetric findings compatible with secondary NET lesions (liver lesions in 3 patients and nodes in 1 patient); in 9 patients, small-sized findings (at lung, nodes, liver, and pancreatic level) were reported as indeterminate on ceCT (4/9 remained stable following imaging).

**Figure 4:**
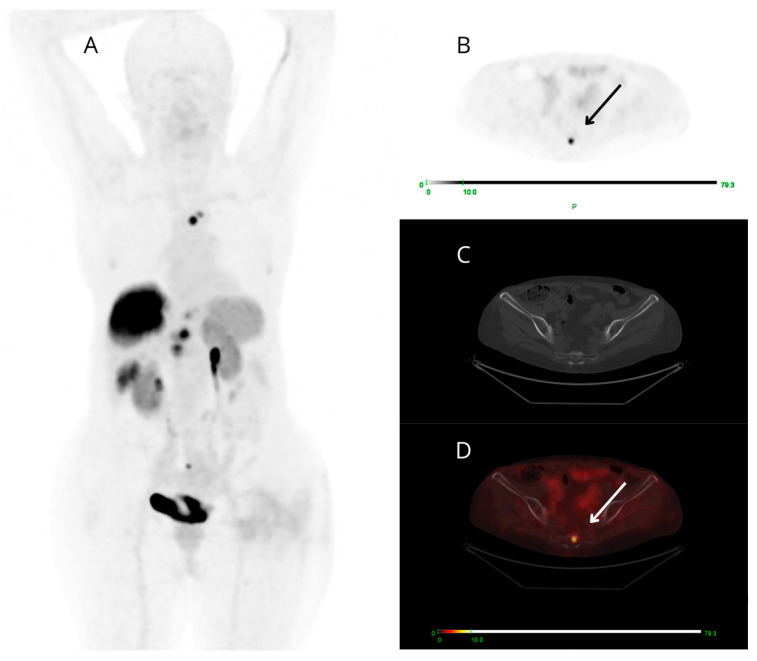
Maximum intensity projection (MIP) (**A**), PET (**B**), CT (**C**), and fused PET/CT (**D**) transaxial images of a patient studied for restaging of metastatic G3 NET, likely of pancreatic origin. [^68^Ga]Ga-DOTANOC PET shows high and focal uptake at the coccyx (arrows). The uptake area does not correspond to clear morphologic alterations on low-dose CT.

Among the 13 PET-positive targeted small findings (all true positive), 6/13 cases presented ceCT-negative small lesions (at bone level in 4/6 patients and node level in 2/6 cases) (Figure 4), 2/13 showed both small-sized PET-positive lesions (false negative on ceCT) and additional small-sized lesions reported as indeterminate on ceCT that were not detectable on PET/CT. There was also a small group of only 5/13 patients presenting both concordant and discordant PET-positive findings: each case presented a target PET-positive small finding confirmed by ceCT (concordant true positive) and a small finding clearly avid on PET images (2 pancreatic, 2 nodal and 1 at soft tissue level), known as malignant at ceCT only after expert radiological revision, aware of the PET result.

## 5. Discussion

PET images are generated using image reconstruction algorithms to improve image quality. Q.Clear was developed to enhance image quality through the improvement of CNR, and it is deemed to increase quantitative accuracy [2]. The superiority of Q.Clear over OSEM was documented in phantom and clinical studies, mostly using [^18^F]F-FDG reconstructed with various β-levels, often between 300 and 400 [4,5,7,8,9,23,24,25]. The optimal β-level in the non-FDG setting was only preliminarily explored in different clinical scenarios. In a study conducted using [^18^F]F-PSMA-1007 PET/CT, the optimal β-level was 700 [26]. In two studies on [^18^F]-NaF, the preferred levels were, respectively, 400 and 600 (the latter for overweight patients) [27,28]. For [^89^Zr]-immunoPET tracers, the best value found was 3600 [29].

Few studies regard Gallium-68 tracers. Santoro et al. analyzed OSEM and Q.Clear reconstructed images of NEMA phantoms filled with Gallium-68 to find the optimal β-level according to lesion size and phantom type (mimicking normal or overweight patients). Optimal β-levels ranged from 250 to 800 [3]. A β-level of 600 was suggested for [^68^Ga]Ga-PSMA PET/CT by Rijnsdrop et al. [30]. Lysvik et al. applied different β-levels to dynamic [^18^F]F-PSMA-1007 PET imaging performed in two patients with recurrent glioblastoma, reporting a 25.5% increase of K_i_ with increased β-levels (from 300 to 1000) [31].

Besides identifying the best reconstruction in terms of overall visual image quality, we conducted additional analysis of ceCT/PET discordant small lesions, expecting improved small-lesion detectability with Q.Clear reconstruction. An improvement in small-lesion detectability in Q.Clear images was previously reported by Macnab et al. In their study, three independent readers blindly compared the visual image quality of active and non-active small objects in OSEM and Q.Clear reconstructions (with a penalization coefficient of 400), finding significant visual-detection improvement in Q.Clear images of active small lesions over OSEM. No significant differences were found for non-active (true-negative) lesions [13]. Miwa et al. recently analyzed the impact on small lesions of another factor of Q.Clear reconstruction, named gamma, whose value of 2 enabled the detection of lesions <6.2 mm in phantoms [32]. Overall, the preferred β-level varies considerably among published studies, also depending on the chosen scan time per bed position. Moreover, semiquantitative measurements are influenced by uptake times and data entries like patient weight, decay-corrected injected dose, and cross-calibration [15].

A potential limitation of this new technology is the occurrence of significant alterations of lesions’ semiquantitative parameters that may hamper the direct comparison of their value across different tomographs and time-points: higher SUV values over OSEM were previously reported for Q.Clear image reconstructions performed using [^68^Ga]Ga-DOTATATE [33], [^18^F]F-FDG [1,7,34], and [^18^F]F-PSMA [35]. Devriese et al. reported statistically significant and clinically relevant differences between Q.Clear (β-factor of 400) and OSEM images in terms of SUVmax and SUVpeak in 64 [^18^F]F-FDG PET/CT examinations [34]. Similarly, Wyrzykowski et al. reported significant discordant clinical assessments (change in Deauville Score) between the two reconstructions in 11 cases (15.7%) of 70 interim [^18^F]F-FDG PET scans and in 11 cases of 70 end-of-treatment PET scans among a total of 280 patients with lymphoma (β-level of 350) [9].

Published studies investigating Q.Clear of patients with neuroendocrine neoplasms undergoing [^68^Ga]Ga-DOTANOC PET/CT are limited. We previously analyzed Q.Clear reconstruction in the subgroup of overweight patients with NEN. In these patients, image quality suffers from low-coincidence events and poor attenuation. Applying the Bayesian penalized-likelihood reconstruction with high β-levels (β1600), the perceived image quality was enhanced, followed by significantly increased CNR, SNR-T, and LSNR [21]. The issue of comparability was also addressed by Krokos et al., who performed PET scans with ^68^Gallium-labelled tracers in 14 patients, reconstructing the images with or without the Q.Clear algorithm. They found that only β-factors of 800–1000 made the two reconstructions comparable in terms of semiquantitative parameters [36].

The present study shows that Q.Clear β1600 reconstruction outperformed STD and β800/β1000 reconstructions. In fact, all readers unanimously agreed that β1600 reconstruction provided the best image quality. When lesion-dependent parameters were analyzed, there were no significant differences between β1600 and STD (regardless of lesion size), confirming that β1600 can be employed in routine clinical practice (allowing the direct comparison of lesions’ semiquantitative parameters even when previous images were acquired using different tomographs). Moreover, the β1600 images present significant differences in SNR liver, resulting in better visualization of lesions at the liver level, the most frequent site of metastatic spread. Limitations of the present study include the small-sized cohort and, in particular, the small subgroup with discordant small lesions. However, it is important to note that the setting of small-lesion detection is challenging with all imaging modalities (ceCT and PET), in particular in the setting of NET, which is characterized by a low growth rate over time. Our data show that all clearly PET-positive target findings <1 cm corresponded to true malignant lesions. In particular, the most frequent sites of small PET-positive/ceCT-negative findings were at the bone level and node level. In the case of a lack of significant uptake on PET corresponding to indeterminate small ceCT findings, clinical and radiological surveillance is recommended.

## 6. Conclusions

Despite the previously mentioned limitations, our data show that when a digital PET/CT is available, β1600 Q.Clear reconstruction for [^68^Ga]Ga-DOTANOC imaging is feasible and improves image quality for both overall and small-lesion assessment. Further studies are needed to validate these findings in a larger population and to assess the impact on patient management derived from the Q.Clear improved image quality.

## Figures and Tables

**Table 1:** Patient epidemiological characteristics.

		Median	Range
Age		62.5	52.7–73.0
Gender		n	%
	Male	31	60
	Female	21	40
Primary tumor site			
	Ileum	15	29
	Pancreas	11	21
	Other GEP	6	11
	Lung	4	8
	CUP	4	8
Suspected NET		12	23
Indication of PET imaging			
	Pre-treatment staging	7	14
	Post-surgical staging	6	11
	Evaluation of PRRT eligibility	2	4
	Suspected relapse	4	8
	Assessment during treatment	8	15
	Restaging after therapy	10	19
	Follow-up	2	4
	Localization of unknown primary	2	4
	Suspected NET	11	21

**Table 2:** Lesion-dependent parameters.

		n	β1600			STD			*p*
			Mean	Median	Range	Mean	Median	Range	
Most avid lesion > 1 cm (T)		37							
	SUVmax		26.7	22.7	4–82.7	26.9	24.3	3.4–79.3	0.867
	SUVmean		16.9	14.1	2.3–54.5	16.6	15.1	2.1–51.7	1000
	CNR		45.6	38.3	2.3–188	35.5	34.8	2.7–125.5	0.165
Most avid lesion < 1 cm		23							
	*concordant*	10							
	SUVmax		10.4	7.5	4.7–24	11.7	8.7	5.1–24.3	0.364
	SUVmean		6.5	4.6	3–15.6	7.0	5.3	3.3–15.1	0.427
	CNR		15.6	5.0	−0.6–60	15.4	5.7	−0.6–49.5	0.734
	*discordant*	13							
	SUVmax		14.8	13.4	7.3–18.4	15.1	11.8	7.8–20.4	0.96
	SUVmean		9.3	8	4.3–11.3	9.7	7.4	4.6–12.5	0.98
	CNR		27.8	18.5	9–32.8	29.6	23.7	10.9–30.7	0.572

## Data Availability

Access to the anonymous electronic archive is regulated by the local ethics committee.
